# TRUmiCount: correctly counting absolute numbers of molecules using unique molecular identifiers

**DOI:** 10.1093/bioinformatics/bty283

**Published:** 2018-04-16

**Authors:** Florian G Pflug, Arndt von Haeseler

**Affiliations:** 1 Center for Integrative Bioinformatics Vienna (CIBIV), Joint Institute of the University of Vienna and Medicial University of Vienna, Max F. Perutz Laboratories (MFPL), Vienna, Austria; 2 Bioinformatics and Computational Biology, Faculty of Computer Science, University of Vienna, Vienna, Austria

## Abstract

**Motivation:**

Counting molecules using *next-generation sequencing* (NGS) suffers from PCR amplification bias, which reduces the accuracy of many quantitative NGS-based experimental methods such as RNA-Seq. This is true even if molecules are made distinguishable using *unique molecular identifiers* (UMIs) before PCR amplification, and distinct UMIs are counted instead of reads: Molecules that are lost entirely during the sequencing process will still cause underestimation of the molecule count, and amplification artifacts like PCR chimeras create phantom UMIs and thus cause over-estimation.

**Results:**

We introduce the TRUmiCount algorithm to correct for both types of errors. The TRUmiCount algorithm is based on a mechanistic model of PCR amplification and sequencing, whose two parameters have an immediate physical interpretation as PCR efficiency and sequencing depth and can be estimated from experimental data without requiring calibration experiments or spike-ins. We show that our model captures the main stochastic properties of amplification and sequencing, and that it allows us to filter out phantom UMIs and to estimate the number of molecules lost during the sequencing process. Finally, we demonstrate that the phantom-filtered and loss-corrected molecule counts computed by TRUmiCount measure the true number of molecules with considerably higher accuracy than the raw number of distinct UMIs, even if most UMIs are sequenced only once as is typical for single-cell RNA-Seq.

**Availability and implementation:**

TRUmiCount is available at http://www.cibiv.at/software/trumicount and through Bioconda (http://bioconda.github.io).

**Supplementary information:**

Supplementary information is available at *Bioinformatics* online.

## 1 Introduction

**Fig. 1.**
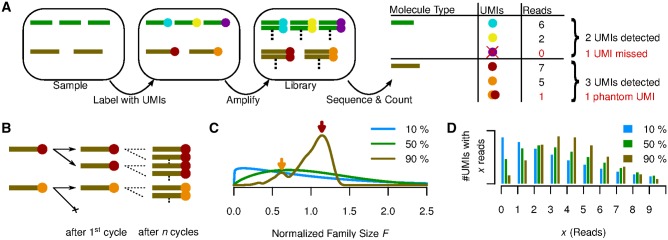
(**A**) The relevant steps of library preparation when the UMI method is used. The sample initially contains three copies of molecule 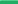 and two copies of 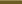, which are made unique by labelling with UMIs (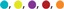). Each of those molecules is expanded into a molecular family during amplification, and a random selection of molecules from these families is sequenced. Counting unique UMIs then counts unique molecules, unless UMIs have read-count zero (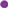) or phantom UMIs are produced (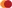). **(B)** PCR as a Galton-Watson branching process. Molecule 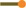 failed to be copied during the first PCR cycle and the final family size is thus reduced compared with 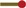. **(C)** Normalized family size distribution for efficiency 10, 50 and 90%. The arrows mark the most likely normalized family sizes for the two molecules from (B), assuming a reaction efficiency of 90%, and taking their distinct fates during the first PCR cycle into account. **(D)** Distribution of reads per UMI for efficiency 10, 50 and 90% assuming D=4 Reads per UMI on average

Experimental methods like RNA-Seq, ChIP-Seq and many others depend on *next-generation sequencing* (NGS) to measure the abundance of DNA or RNA molecules in a sample. The PCR amplification step necessary before sequencing often amplifies different molecules with different efficiencies, thereby biasing the measured abundances (Aird *et al.*, 2011). This problem can be alleviated by ensuring that all molecules are distinguishable before amplification by some combination of factors comprising a *unique molecular identifier* (UMI) (Hug and Schuler, 2003; Kivioja *et al.*, 2012), which usually includes a distinct molecular barcode ligated to each molecule before amplification (Fig. 1A, colored dots; see Smith *et al.* (2017) for a more extensive history of the UMI method). After amplification and sequencing, instead of counting reads, reads are grouped by UMI, and each distinct UMI is taken to reflect a distinct molecule in the original sample (Fig. 1A). But while the number of distinct UMIs may be a better proxy for the molecule count, it is still biased, for two reasons:

- Molecules that are amplified with low efficiency will have fewer copies made, hence fewer reads per UMI, and thus a higher chance of being left entirely unsequenced (Fig. 1A, green transcript, violet UMI).
- Sequencing errors, PCR chimeras, and index miss-assignment (Sinha *et al.*, http://www.biorxiv.org/content/early/2017/04/09/125724) in multiplexed sequencing runs can produce *phantom UMIs* which do not correspond to any molecule in the original sample (Fig. 1A, orange/red phantom UMI).

Various methods have been proposed to counter-act these effects: Smith *et al.* (2017) proposed an algorithm for merging highly similar, erroneous versions of the same original UMI to correct for sequencing errors and single-nucleotide PCR amplification errors. To filter out more complex PCR artifacts, strand-specific UMI-labeling protocols were introducted (Shiroguchi *et al.*, 2012; Schmitt *et al.*, 2012) that allow filtering out artifacts based on whether UMIs for both strands of a template molecule were detected. A correction for molecules left entirely unsequenced is mentioned by Kivioja *et al.* (2012), but being based on the Poission distribution, it severely under-estimates the amount of affected molecules; for their data by about an order of magnitude.

Instead of relying on sequence similary or complicated strand-specific UMI-labeling protocols, we rely on the per-UMI read count to separate *true UMIs* (i.e. UMIs of actual molecules in the original sample) from *phantom UMIs*. Chimeric PCR products are typically produced during later reaction cycles, and can therefore be expected to have smaller copy numbers and hence a lower read-count than non-chimeric PCR products. Index miss-assignment and sequencing errors typically happen randomly, and are unlikely to produce a larger number of reads showing the same phantom UMI. For these reasons, phantom UMIs can be expected to have a markedly lower read count than most *true UMIs*, i.e. UMIs of actual molecules in the original sample.

Our bias-correction and phantom-removal algorithm TRUmiCount exploits this difference in expected read counts between phantoms and true UMIs. It removes UMIs likely to be phantoms based on a read-count threshold, and then estimates and corrects the (gene-specific) loss, i.e. the fraction of molecules that were not sequenced or whose UMIs were mistaken for phantoms. For this correction TRUmiCount employs a model of PCR amplification that accounts for the stochasticity inherent to this amplification reaction.

## 2 Materials and methods

### 2.1 The TRUmiCount algorithm

The TRUmiCount algorithm consists of the following three steps:

1. We first filter out phantom UMIs by removing any UMI whose read count lies below a suitably chosen *error-correction threshold* (*T*).
2. We then estimate the loss (ℓ), i.e. the fraction of molecules that were not sequenced at all, or whose UMIs were removed by the error-correction threshold. This estimate is computed using a stochastic model of the amplification and sequencing process whose parameters are the PCR efficiency (*E*), and the sequencing depth (*D*), expressed as the average number of reads per UMI in the initial sample. From the observed distribution of reads per UMI, we estimate both (raw) gene-specific as well as library-wide values for these parameters, and compute corresponding estimates of the loss (see Section 2.2 for details).
3. Finally, we add the estimated number of lost UMIs back to the the observed number of true UMIs (those UMIs with ≥ threshold reads) to find the total number of molecules in the original sample. Since the loss can vary between genes, to yield unbiased counts, the correction must be based on gene-specific loss estimates. Due to the noise inherent to raw gene-specific estimates for genes with only few observed true UMIs, we employ a James-Stein-type (James and Stein, 1961) *shrinkage estimator*, adjusting the raw gene-specific parameter and loss estimates towards the library-wide ones (thus *shrinking* their difference). We choose the amount of shrinkage based on each estimate’s precision, in such a way that the expected overall error is minimized (Carter and Rolph, 1974) (see Section 2.3).

### 2.2 Estimating the fraction of lost molecules

To estimate the loss, i.e. the fraction of molecules whose UMIs had a read count below the error-correction threshold, we model the distribution of per-UMI read counts by combining a stochastic model of PCR amplification with a model of NGS as random sampling.

#### 2.2.1 A stochastic model of PCR amplification

To model PCR amplification, we use the *single-stranded* model of Krawczak *et al.* (1989), meaning we view PCR as a stochastic process that during each cycle duplicates each molecule independently with a particular probability *E*, called the reaction’s *efficiency*. We further assume that a molecule is copied perfectly or not at all, i.e. that neither partial copies nor copies with a slightly different base-pair sequence are produced, that no molecules are destroyed or lost, and that the efficiency *E* stays constant throughout the reaction. Although this model has been extended by Weiss and von Haeseler (1997) to include the possibility of substitution errors during amplification, exhaustively modeling *all* possible sources of phantom UMIs seems futile. We therefore pursue a different approach, and model only the error-free case, trusting the error-correction threshold to remove phantoms. Over multiple cycles, each molecule is thus assumed to be expanded into a *molecular family* of identical copies. Since we use the single-stranded model, *molecule* for us always means a single-stranded piece of DNA, and we do not distinguish between a strand and its reverse complement. For our purposes, a piece of double-stranded DNA thus consists of two indistinguishable molecules.

Before amplification, we assume all molecules in the sample to be distinguishable by some UMI. During amplification, each of those molecules gives rise to a *molecular family* of (indistinguishable) copies. The initial size of such a family (i.e. the number of copies it is comprised of) is 1. During the first PCR cycle, the size increases to 2 if the single initial molecule is copied successfully, i.e. with probability *E*. Continuation of this process, always using all existing molecules as potential templates that are copied with probability *E*, produces a random sequence $M_{0},M_{1},M_{2},\ldots$ of molecular family sizes after the 0th, 1st, 2nd, … cycle. This sequence forms a Galton-Watson branching process (Weiss and von Haeseler, 1995), and follows the recursion

(1) $$\begin{array}{c} M_{0}=1,\;M_{i}=M_{i-1}+\Delta_{i}\;\text{where}\\ \Delta_{i}\;∼\;\text{Binom}\;(M_{i-1},E). \end{array}$$

Although we are not aware of a way to obtain an explicit formula for the distribution of the family size *M_i_* after *i* PCR cycles, the expected value and variance of *M_i_* can be computed explicitly. According to Harris (1989, Ch. 1), Equation (5.3), $VM_{i}=\frac{\sigma^{2}m^{i}(m^{i}-1)}{m^{2}-m}$ where *m* and *σ* are the mean and SD of *M*_1_. In our case these are m=1+E and $\sigma^{2}=E\cdot (1-E)$, thus we find

(2) $$EM_{i}={\left(1+E\right)}^{i}$$

(3) $$VM_{i}=\frac{1-E}{1+E}\cdot {\left(1+E\right)}^{i}({\left(1+E\right)}^{i}-1)$$

Equation 2 shows the well-known exponential growth of expected family sizes during PCR. But apart from recovering this well-known property of PCR, the Galton-Watson model also predicts the likelihood of *deviations* from this expectation due to random failures of copy operations, and by simulation allows us to find the actual distribution of *M_i_*.

#### 2.2.2 The normalized family size *F*

Due to the exponential growth of the expectation of *M_i_*, the distribution of *M_i_* depends heavily on the PCR cycle count *i*. That dependency, however, affects mostly the *scale*, not the *shape* of the distribution of *M_i_*. To see the effect on the shape more clearly, the effect on the scale is removed by replacing *M_i_* with a re-scaled version which has an expected value of one,

(4) $${\tilde{M}}_{i}=\frac{M_{i}}{EM_{i}}=\frac{M_{i}}{{\left(1+E\right)}^{i}}.$$

These re-scaled family sizes can be sensibly compared across PCR cycles. We observe that with growing cycle counts, the additional stochasticity introduced by each additional cycle drops rapidly. The re-scaled family size after the first cycle varies by a factor of two depending on whether the (single) copy operation during the first cycle succeeds or fails. Later on there are more templates to copy from, and thus the success or failure to copy any particular molecule averages out, making the behavior of the reaction more deterministic. Finally, ${\tilde{M}}_{i}\approx {\tilde{M}}_{i+1}$, because the family size *M_i_* increases during each cycle almost exactly by a factor of 1+E, which matches the decrease of the re-scaling factor in ${\tilde{M}}_{i}$. This informal argument can be turned into a formal proof (see Harris (1989), Ch. 1, Th. 8.1) of the convergence of the re-scaled family size as *i* tends towards ∞, which allows us to remove the cycle count as a parameter entirely from what we call the *normalized family size*

(5) $$F=\underset{i\to \infty }{\lim}{\tilde{M}}_{i}.$$

Although there is again no explicit formula known for the distribution of the normalized family size *F*, we find its variance from Equations (3–5) to be

(6) $$VF=\frac{1-E}{1+E}.$$

To quickly evaluate the density $f_{F}(x;\;E)$ of the distribution of *F* for a particular normalized family size *x* given reaction efficiency *E*, we interpolate using 2D polynomial interpolation (Akima, 1996) between pre-computed densities for different reaction efficiencies between 0 and 100% at different family sizes between 0 and 50 (see Supplementary Section S1.1 for details).

#### 2.2.3 Modeling the sequencing process

The normalized family size distribution models the abundance of molecules with a particular UMI. To model the read count of a particular UMI after sequencing (i.e. the number of reads stemming from a particular pre-amplification molecule), we model NGS with a Poissonian sampling model (Marioni *et al.*, 2008). This amounts to assuming that (i) each individual copy has the same probability of being sequenced, (ii) this probability is small compared to the sequencing depth and (iii) there were many (distinguishable) original molecules. We further assume that a UMI is *on average* represented by *D* reads. Then the read count *C* of a UMI with known normalized molecular family size *F* is Poisson distributed,

(7) $$\begin{array}{c} C\;|\;F∼\text{Poisson}\;(F\cdot D),\\ P(C=k\;|\;F)=e^{-F\cdot D}\frac{{\left(F\cdot D\right)}^{k}}{k!}. \end{array}$$

In general, however, the exact family size *F* of any particular UMI is unknown—we only know the *distribution* of *F*. To compute the probability of a UMI having *k* reads, we average over all possible family sizes x∈[0,∞), weighting them with their respective density $f_{F}(x;\;E)$ in the distribution of the normalized family size *F*,

(8) $$P(C=k)=\int_{0}^{\infty }P(C=k\;|\;F=x)\cdot f_{F}(x;\;E)\;dx.$$

We note that while P(C=k) depends on *D* and *E*, we omit these dependencies for brevity of notation. To compute the probabilities P(C=k), we integrate numerically using the midpoint rule on the grid of family sizes *x* for which $f_{F}(x;\;E)$ was pre-computed. For the mean and variance of *C* we find the explicit expressions

(9) $$E(C)=D,\;V(C)=D+D^{2}\frac{1-E}{1+E}.$$

Since we impose an error-correction threshold *T* and drop UMIs with fewer than *T* reads, the read-count distribution we actually observe is a *censored* version of *C* where the possible outcomes *C *<* T* are removed. For the mean and variance of this censored distribution with threshold *T* we write

(10) $$E(C\;|\;C\geq T)=\frac{\sum_{k=T}^{\infty }k\cdot P(C=k)}{P(C\geq T)},$$

(11) $$V(C\;|\;C\geq T)=\frac{\sum_{k=T}^{\infty }(k-E(C\;|\;C\geq T){)}^{2}\cdot P(C=k)}{P(C\geq T)}.$$

To compute E(C | C≥T), we rewrite the infinite sum in Equation (10) to $E(C)-\sum_{k<T}k\cdot P(C=k)$, and similarly for V(C | C≥T).

#### 2.2.4 Computing the loss

The expected loss ℓ is the expected fraction of true UMIs that either remain completely unsequenced, or that are removed by the error-correction threshold. Since we treat each per-UMI read count, and hence each UMI’s fate (to be filtered or not) as independent stochastic quantities, this expected fraction is simply the probability that a single UMI has a read-count below the threshold *T*, i.e.

(12) ℓ=P(C<T).

### 2.3 Correcting for lost molecules

Given $n^{\text{obs}}$ experimentally observed UMIs (after applying the error-correction threshold *T* to filter out phantoms) and their read count vector $c=(c_{1},\ldots ,c_{n^{\text{obs}}})$, we estimate the reaction efficiency *E* and the mean number of reads per UMI *D*. We use the *method of moments*, i.e. we find *E* and *D* such that the predicted mean equals the sample mean $\hat{m}$ of **c**, and the predicted variance its sample variance $\hat{v}$. Since we only take observed UMIs with at least *T* reads into account, we must compute the predictions using the censored distribution, i.e. find *E*, *D* such that $\hat{m}=E(C\;|\;C\geq T)$ and $\hat{v}=V(C\;|\;C\geq T)$

If *T* = 0, i.e. if $\hat{m}$ and $\hat{v}$ reflect the *uncensored* mean respectively variance, these equations can be solved explicitly by inverting Equation (9), which yields the method of moments estimates $\hat{D}=\hat{m}$ and $\hat{E}=\frac{1-v\prime }{1+v\prime }$, where $v\prime =\frac{\hat{v}-\hat{m}}{{\hat{m}}^{2}}$ limited to the interval [0,1].

If *T* > 0, we solve the system of equations numerically to find *E* and *D* (see Supplementary Section S1.2). With these parameter estimates, we then compute an estimate $\hat{\ell }$ of the loss ℓ using Equation (12), and use it to correct for the expected number of lost molecules. Assuming that we observed $n^{\text{obs}}$ UMIs and given $\hat{\ell }$, we estimate the total number of molecules in the original sample to have been

(13) $${\hat{n}}^{\text{tot}}=\frac{n^{\text{obs}}}{1-\hat{\ell }}.$$

#### 2.3.1 Gene-specific estimates and corrections

Since the reaction efficiency *E* and depth *D*, and hence also the loss, will usually vary between individual genes (or other genomic features of interest), to correct the observed number of transcripts of some gene g∈1,…,K for the loss, a *gene-specific* loss estimate ${\hat{\ell }}_{g}$ should be used. In principle, such estimates are found by applying the described estimation procedure to only the UMIs found for transcripts of gene *g*, i.e. by computing a gene-specific mean ${\hat{m}}_{g}$ and variance ${\hat{v}}_{g}$ of the number of reads per UMI, and solving for parameters *E* and *D* to find a gene-specific ${\hat{E}}_{g}^{\text{raw}}$ and ${\hat{D}}_{g}^{\text{raw}}$, and computing ${\hat{\ell }}_{g}^{\text{raw}}$ using Equation (12). If the number $n_{g}^{\text{obs}}$ of observed UMIs (i.e. transcripts) stemming from gene *g* is large, a correction based on ${\hat{\ell }}_{g}^{\text{raw}}$ yields an (approximately) unbiased and accurate estimate of the total number of transcripts of that gene. But if $n_{g}^{\text{obs}}$ is small, the error of the estimator ${\hat{\ell }}_{g}^{\text{raw}}$ easily exceeds the variability of the true gene-specific value $\ell_{g}$ between genes. In such cases, correcting using the *library-wide* estimate ${\hat{\ell }}^{\text{all}}$ computed from *all* UMIs found in the library will yield a more accurate (although biased) estimate of the total number transcripts of gene *g*.

Interestingly, by combining these two flawed estimators of the true gene-specific loss $\ell_{g}$, we obtain a *shrinkage estimator*${\hat{\ell }}_{g}^{\text{shr}}$ that improves upon both in terms of *mean squared error* (MSE), see Carter and Rolph (1974) Equation (2.4),

(14) $${\hat{\ell }}_{g}^{\text{shr}}=\lambda_{g}\cdot {\hat{\ell }}_{g}^{\text{raw}}+(1-\lambda_{g})\cdot {\hat{\ell }}^{\text{all}}.$$

The gene-specific coefficient *λ_g_* determines how much the raw gene-specific estimate is *shrunk* towards the global estimate, and its optimal choice (with respect to the MSE) depends on the variances the two constituent estimators. To determine the optimal *λ_g_* we make the following assumptions about these estimators:

- the library-wide estimate ${\hat{\ell }}^{\text{all}}$ is a good proxy for the true *average* loss taken over all genes 1,…,K. This seems reasonable given the size of a typical library, comprising millions of UMIs.
- the estimator variance of the raw gene-specific estimator ${\hat{\ell }}_{g}^{\text{raw}}$ depends only on the number $n_{g}^{\text{obs}}$ of observed UMIs for gene *g*, and does so in an inversely proportional manner. This is certainly true asymptotically for large numbers of observations, for small numbers Supplementary Figure S4 shows this approximation to be reasonable.

We write *s* for the variance of the true loss between genes (i.e. for the mean squared difference of $\ell_{g}$ and ${\hat{\ell }}^{\text{all}}$), and *u* for the proportionality constant between the estimator variance of ${\hat{\ell }}_{g}^{\text{raw}}$ and $1/n_{g}^{\text{obs}}$. According to Carter and Rolph (1974)Equation (2.4ff) the optimal choice for *λ_g_* is then

(15) $$\lambda_{g}=\frac{s}{s+u/n_{g}^{\text{obs}}}$$

To compute the gene-specific shrinkage estimators ${\hat{\ell }}_{g}^{\text{shr}}$, it remains to find constants *u* and *s*. Towards that end, we observe that the expected squared deviation of the raw gene-specific loss estimate ${\hat{\ell }}_{g}^{\text{raw}}$ from its average $\bar{\ell }=\frac{1}{n}\sum_{g=1}^{K}{\hat{\ell }}_{g}^{\text{raw}}$ is the total variance of ${\hat{\ell }}_{g}^{\text{raw}}$, which is comprised of the between-gene variance *s* and the estimator variance $u/n_{g}^{\text{obs}}$, or in other words $E\;{\left({\hat{\ell }}_{g}^{\text{raw}}-\bar{\ell }\right)}^{2}=s+u/n_{g}^{\text{obs}}$.

This allows us to estimate *s* and *u* using *least squares regression*, i.e. by minimizing

(16) $$\sum_{g=1}^{K}({\left({\hat{\ell }}_{g}^{\text{raw}}-\bar{\ell }\right)}^{2}-s-u/n_{g}^{\text{obs}}{)}^{2}\cdot w(n_{g}^{\text{obs}}).$$

Without weighting (i.e. for *w*(*n*) = 1), the considerable drop in magnitude of ${\left({\hat{\ell }}_{g}^{\text{raw}}-\bar{\ell }\right)}^{2}$ as $n_{g}^{\text{obs}}$ increases would allow genes with small number of observations to yield an unduly large influence over the estimates. Since it is the genes with a low to moderate number of observations that benefit from shrinking, some modest bias of this sort is actually desired—but not as strong a bias as *w*(*n*) = 1 exhibits, and one not so purely focused on genes with very few observations. We therefore use the weights $w(n)=\frac{n}{1+n/100}$, which initially increase linearly with the number of observations, but eventually converge to 100 instead of increasing further. This has the desired effect of shifting the focus away from rarely observed genes, and concentrating it on genes with a moderate number of observations.

## 3 Results

### 3.1 PCR stochasticity versus efficiency

During PCR amplification, each uniquely labeled molecule is amplified into a molecular family of indistinguishable copies. Random successes or failures to copy molecules during early reaction cycles lead to a variation in the final family sizes (Fig. 1B), even between identical (expect for their molecular barcode) molecules. As the family size of each initial molecule grows, the proportion of successful copy operations approaches the efficiency *E*, therefore reducing the amount of noise added by each additional cycle. The total number of cycles thus has little influence on the final family size distribution, and is therefore not a parameter of our model. For the same reason, a plateau effect (i.e. diminishing reaction efficiency during later cycles) has little effect on the final family size distribution, and is thus not included in the model. The final distribution does, however, depend strongly on the reaction efficiency, with fluctuations in family size decreasing as the efficiency grows towards 100% (Fig. 1C).

For efficiencies close to 100%, most molecular families are thus of about average size, except for those (∼100−E percent) families for which the first copy failed. These are about half the average size, and form a distinct secondary peak in the family size distribution (Fig. 1C, brown curve). We emphasize that due to this, even at efficiencies close to 100%, the distribution still shows considerable dispersion, meaning that even at high efficiencies stochastic PCR effects are not negligible. At lower efficiencies, the family sizes vary even more wildly, as extreme family sizes (on both ends of the scale) become more likely (Fig. 1C, blue and green curves).

If we add sequencing to the picture, i.e. combine the stochastic PCR model outlined above with a model of sequencing as random Poissonian sampling (Marioni *et al.*, 2008), the variability of per-UMI read counts (Fig. 1D) then has two sources—the variability of molecular family sizes and the Poissonian sampling introduced by sequencing. Although the latter is reduced by increasing the sequencing depth, the former is independent of the sequencing depth but is reduced by increasing the reaction efficiency. For all reasonable error-correction thresholds *T* the predicted fraction of true UMIs filtered out by the error-correction step thus grows with diminishing efficiency *E*.

### 3.2 Model validation and phantom UMI removal

To validate our model of amplification and sequencing, we compared the predicted distribution of per-UMI read counts to the distribution observed in two published RNA-Seq datasets. Kivioja *et al.* (2012) labeled and sequenced transcripts in *Drosophila melanogaster* S2 cells using 10 bp random molecular barcodes from the 5′ end. Shiroguchi *et al.* (2012) labeled and sequenced transcript fragments in *E.coli* cells on both ends, using (on each end) one of 145 molecular barcodes carefully selected to have large pairwise edit distances. The Y-shaped sequencing adapters used in the *E.coli* experiment were designed such that each strand of a labeled double-stranded cDNA molecule produces a related but distinguishable molecular family.

To see whether our algorithm offers an advantage over existing UMI error-correction strategies, we pre-filtered the observed UMIs in each of the two replicates of these datasets using the following existing algorithms: We first merged UMIs likely to be erroneously sequenced versions of the same molecule, using the algorithm proposed by Smith *et al.* (2017). For the *E.coli* experiment we also removed UMIs for which the complementary UMI corresponding to the second strand of the same initial template molecule was not detected, as proposed by Shiroguchi *et al.* (2012). See Supplementary Section S1.4 for details on the analysis pipeline we used.

**Fig. 2.**
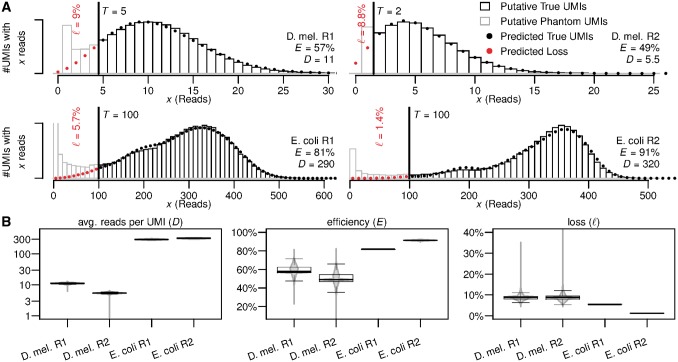
(**A**) Observed and predicted library-wide distribution of reads per UMI and parameter and loss estimates. Filtered UMIs (grey bars, left of threshold *T*) are over-abundant and thus assumed to contain both phantom and true UMIs (red dots). UMIs surviving the filter (black bars) closely follow the predicted distribution (black dots) and are assumed to be true UMIs. **(B)** Variability of the (shrunken) model parameters and resulting loss between genes. Includes parameter for 7481 detected genes in *D.melanogaster* R1, 8001 genes in R2, 2380 genes in *E.coli* R1 and 2308 genes in R2. (Color version of this figure is available at *Bioinformatics* online.)

To this pre-filtered set of UMIs we then applied our algorithm. For each dataset, we manually chose an error-correction threshold by visually comparing read-count distribution and model prediction for different thresholds, and picking the lowest threshold that yielded a reasonably good fit. Above the error-correction threshold (Fig. 2a, black bars), the observed library-wide distribution of reads per UMI closely follows the model prediction, and the *E.coli* data even shows traces of the secondary peak that represents molecules not duplicated in the first reaction PCR cycle. Choosing a different threshold will change the number of UMIs surviving the error-correction filter, but has little influence on the estimated reaction efficiency and on the estimated total number of UMIs after loss correction (Supplementary Fig. S1). We thus conclude that our model captures the main stochastic behavior of the amplification and sequencing processes, and accurately models the read-count distribution of true UMIs.

The UMIs removed by our filter, i.e. those with fewer reads than the error-correction threshold demands, (Fig. 2A, gray bars) are over-abundant compared to our prediction. This over-abundance increases further as per-UMI read counts drop, indicating the existence of a group of UMIs with significantly reduced molecular family sizes. While we may expect some systematic variation of family sizes between true UMIs (on top of the stochastic variations that our PCR model predicts), we would expect these to be gradual and not form distinct groups. We conclude that the extra UMIs causing the observed over-abundance are indeed phantoms that are rightly removed by our algorithm. We note that none of these phantoms were removed by either the UMI merging algorithm of Smith *et al.* (2017), or (for the *E.coli* data) by filtering UMIs for which the complementary UMI (representing the second strand of the template molecule) was not detected.

For the *D.melanogaster* data, our loss estimates of 9% (R1) and 8.8% (R2) are about a magnitude higher than the 1% (R1) and 2% (R2) estimated using the (truncated) Poisson distribution suggested by Kivioja *et al.* (2012). Given that using a Poisson model amounts to assuming a 100% efficient duplication of molecules during each PCR cycle, this severe underestimation by the Poisson model shows that the inherent stochasticity of the PCR cannot be neglected.

### 3.3 Gene-specific quantification bias

The gene-specific (shrunken) estimates for amplification efficiency, average reads per UMI, and loss that our algorithm produces, vary between genes to different degrees (Fig. 2B). We observe the smallest amount of variation for the average number of reads per UMI (Fig. 2B, left)—the estimates of this parameter are virtually identical for a large majority of genes, and differs only for a few outliers.

The estimated amplification efficiencies on the other hand can vary substantially between genes (Fig. 2B, middle). For the two *D.melanogaster* replicates the range is 22–81% (R1) and 1–83% (R2). Considering that in this experiment only the 3’ ends of transcripts were sequenced, and all fragments contributing to a gene hence share a similar sequence composition, this is not unexpected. These differences in efficiency cause the loss to vary heavily between genes as well (Fig. 2B, right), between 4 and 35% for R1 and between 4 and 89% for R2 (which has a much lower overall sequencing depth). Without gene-specific loss corrections, abundance comparisons between genes will thus suffer from systematic quantification bias against certain genes of up to ≈ 35–4% = 31% for R1 and up to ≈ 85% for R2. The larger amount of systematic bias in *D.melanogaster* R2 is caused by the two-fold reduction of the number of reads per molecules in R2 compared with R1—due to the lower number of reads per molecule in R2, the same difference in amplification efficiencies between two genes translates into a larger difference of the number of lost molecules in R2 compared with R1.

In contrast, fragments from all parts of the transcript were sequenced in the *E.coli* experiments, and together with the high sequencing depth (≈300 reads per UMI), we now expect little variations of efficiency, and small and highly uniform losses across genes. Our efficiency and loss estimates reflects this (Fig. 2B, middle and right), and as the lack of outliers shows, they do so even for genes with only few UMIs. Yet for these genes, the raw (unshrunken) gene-specific estimates are noisy (Supplementary Fig. S3), proving that shrinking the raw estimates successfully reduces the noise to acceptable levels.

### 3.4 Bias-corrected transcript counts

To further verify the accuracy of the corrected transcript counts computed by our algorithm, we conducted a simulation study. We use the (loss-corrected) estimated total transcript abundances of *D.melanogaster* replicate 1, rounded to 10, 30, 100, 300, 1000, 3000 or 10 000 molecules as the true transcript abundances. We then simulated amplification and sequencing of these transcripts, using for each gene the previously estimated gene-specific efficiency and average number of reads per UMI (Fig. 2B). To the resulting list of UMIs and their read-counts for each gene we applied our algorithm to recover the true transcript abundances (threshold *T* = 5 as before), and determined for each gene the relative error of the recovered abundances compared with the simulation input.

**Fig. 3.**
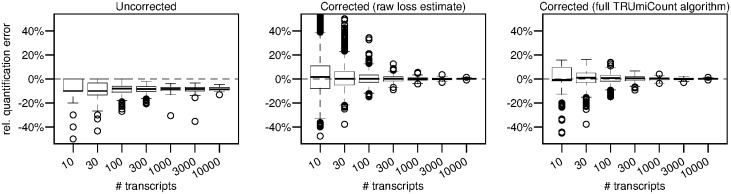
Relative error of estimated total number of transcripts depending on the true number of transcripts. Left panel uses the observed number of UMIs without any correction. Middle panel uses the raw gene-specific loss estimates to correct for lost UMIs. Right panel uses the full TRUMmiCount algorithm employing shrunken gene-specific loss estimates to correct for lost UMIs

Figure 3 shows these relative errors (i) if no correction is done (ii) if the correction is based soley on the raw gene-specific loss estimates (i.e. no shrinkage) and (iii) for the full TRUmiCount algorithm (i.e. using shrunken loss estimates). The uncorrected counts systematically under-estimate the true transcript counts, in 50% of the cases by at least ≈10%, independent of the true number of transcripts per gene. And even at high transcript abundances, the relative error still varies *between* genes, biasing not only absolute transcript quantification, but also relative comparisons between different genes. The counts corrected using raw gene-specific estimates are unbiased and virtually error-free for strongly expressed genes, but exhibit a large amount of additional noise for weakly expressed genes. The full TRUmiCount algorithm successfully controls the amount of added noise, and shows no additional noise for weakly expressed genes, while still being unbiased and virtually error-free for more strongly expressed genes.

### 3.5 Peformance for low sequencing depth

**Fig. 4.**
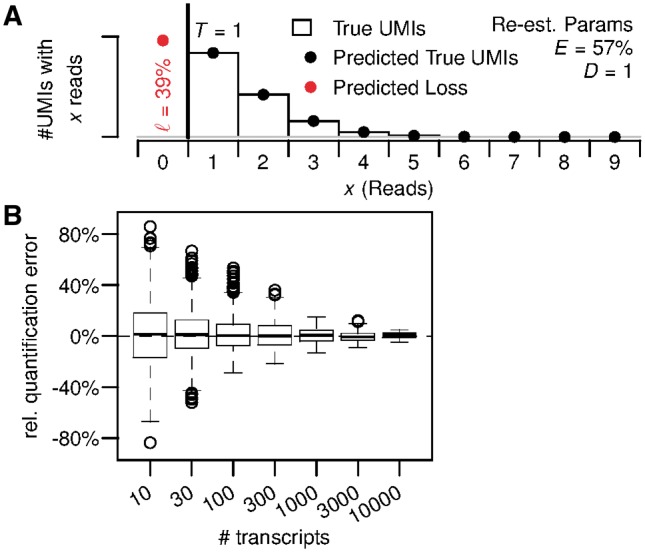
TRUmiCount performance for low sequencing depth. **(A)** Overall distribution of observed and predicted reads per UMI for an average of *D* = 1 read per molecule. **(B)** Relative error of estimated total number of transcripts for different true numbers of transcripts and *D* = 1 read per molecules on average. (Color version of this figure is available at *Bioinformatics* online.)

To assess the performance of the TRUmiCount algorithm at low sequencing depths such as are common for single-cell RNA-Seq experiments, we ran a second simulation with gene-specific depth parameters scaled such that the average across all genes was *D* = 1 read per molecule (Fig. 4). Under these conditions, the most likely outcome for a single molecule in the initial sample is to remain unsequenced (39% of molecules), and only 27% of molecules are found in more than one read. The library-wide efficiency estimate of 57% (Fig. 4A) is nevertheless accurate, and identical to the one computed for the full dataset (*D.melanogaster* R1) that the simulation was based on (Fig. 2A).

For the relative error of the corrected transcript counts we observed a roughly 2-fold increase at low-sequencing depth (Fig. 4B) compared with the situation at original sequencing depth (Fig. 3, right), but still no systematic over- or under-estimation. We estimate that Poissonian sampling effects account for about a $\sqrt{1-0.09}/\sqrt{1-0.39}\approx 1.2$-fold increase of the relative errors. The rest is probably due to the parameter estimation problem becoming harder at lower sequencing depths, particularly for weakly expressed genes. For more strongly expressed genes, the relative quantification error again drops towards zero, similar to the behavior at original sequencing depth.

## 4 Discussion

The TRUmiCount algorithm we presented successfully removes the biases inherent in raw UMI counts, and produces unbiased and low-noise measurements of transcript abundance, allowing for unbiased comparisons between different genes, exons and other genomic features. It does so even in the presence of various types of phantom UMIs and varying amplification efficiencies, both between samples and along the genome. Compared to other error-correction techniques, it is not restricted to particular types of phantom UMIs, or to a special Y-shaped design of the sequencing adapters.

Our model of the amplification and sequencing process is mechanistic, and its two parameters have an immediate physical interpretation. They can both be determined from the experimental data without the need for either guesses or separate calibration experiments. The TRUmiCount algorithm thus does not require any changes to library preparation over the basic UMI method. By inspecting the estimated parameters—in particular the amplification efficiency, the amplification reaction itself can be studied. For example, by estimating model parameters separately for sequenced fragments of different lengths, the drop of reaction efficiency with increasing fragment lengths can be quantified (Supplementary Fig. S2).

Although TRUmiCount requires that libraries are sequenced sufficiently deeply to detect at least some UMIs more than once, it can also deal with cases where a molecule is on average detected only by a single read, which is common e.g. for single-cell RNA-Seq. The performance of TRUmiCount is reduced a bit in such situations, but it still offers an improvement over uncorrected counts by removing systematic biases. For even lower read counts, where gene-specific bias correction becomes infeasible, we expect that TRUmiCount could still be used to correct for cell-specific (instead of gene-specific) biases, thus reducing the amount of technical noise when comparing absolute transcript counts of the same gene between individual cells.

The TRUmiCount algorithm can thus help to increase the accuracy of many quantitative applications of NGS, and by removing biases from comparisons between genes can aid in the quantitative unraveling of complex gene interaction networks. To make our method as easily accessible as possible to a wide range of researchers, we provide two readily usable implementations of our algorithm. Our R package *gwpcR* enables a flexible integration into existing R-based data analysis workflows. In addition, we offer the command-line tool TRUmiCount which is designed to work in conjunction with the *UMI-Tools* of Smith *et al.* (2017). Together they provide a complete analysis pipeline which produces unbiased transcript counts from the raw reads produced by a UMI-based RNA-Seq experiment (http://www.cibiv.at/software/trumicount).

## Supplementary Material

Supplementary Data
