# Supplementary material for: TRUmiCount: correctly counting absolute numbers of molecules using unique molecular identifiers
Source: Bioinformatics. 2018 Apr 16;34(18):3137–44. doi: 10.1093/bioinformatics/bty283 (PMC6157883; doi:10.1093/bioinformatics/bty283)
Supplement: Supplementary Data [file bty283_supplement.pdf]

TRUmiCount: Correctly counting absolute numbers of molecules using unique molecular identifiers

Supplementary Information

Florian G. Pflug and Arndt von Haeseler

S1 Supplementary Methods

S1.1 Computing the distribution of F

To find the actual distribution (in terms its density $f_F(\cdot\,;\,E)$ with the reaction efficiency E as a parameter) of the normalized family size F for a particular efficiency E we resorted to simulation. We simulated the PCR process for efficiencies from 0.01 to 0.99 (steps of 0.01 up to 0.90, steps of 0.005 up to 0.94, steps of 0.002 up to 0.99). Each time, we simulated 10^9 independent trajectories, and ran each simulation until the expected family size was 10^7 molecules (i.e. for $n = 7/\log_{10}(1+E)$ cycles). At that point the stochasticity further cycles would introduce is negligible and we may thus assume $\tilde{M}_n \approx \tilde{M}_{n+1} \approx F$.

For each efficiency E, we normalized the simulated raw family sizes using Equation (4) to obtain 10^9 independent samples of F. Using kernel density estimation, we then estimated values of the density function $f_F(\lambda\,;\,E)$ of the normalized family size distribution on a grid of 318 values of λ between 0 and 50. The grid points are spaced non-uniformly, being finest (distance 0.0025) around 0 and 1 and getting coarser elsewhere.

This procedure resulted in a 123×318 matrix of densities, i.e. $f_F(\lambda\,;\,E)$ evaluated for each combination of one of the 123 simulated efficiencies E, and one of the 318 normalized family sizes λ. Using this (pre-computed and stored) matrix, the density function $f_F(\lambda\,;\,E)$ can be evaluated quickly for arbitrary values of E and λ by two-dimensional polynomial interpolation (Akima, 1996).

S1.2 Numerical method of moments estimates

To obtain method of moments estimates for model parameters D (reads per molecules) and E (reaction efficiency) in the general case $T \geq 0$ from the observed mean $\hat{m}$ and observed variance $\hat{v}$ of the number of reads per UMI, we must find D and E such that

$$\hat{m} = \mathbb{E}(C \,|\, C \geq T),$$
$$\hat{v} = \mathbb{V}(C \,\big|\, C \geq T)$$

We solve this system of equations with an iterative method that starts with initialization step I and then repeats update step U until the estimates $\hat{D}$, $\hat{E}$ and $\mathbb{P}(C \geq T)$ converge (absolute or relative change less than 10^{-4}).

I: We start by pretending that $T = 0$, and set

$$\hat{D} := \hat{m},$$
$$\hat{E} := \frac{1-r}{1+r} \quad \text{where} \quad r = \frac{\hat{v} - \hat{m}}{\hat{m}^2} \quad \text{limited to } [0,1],$$

U: Using the current model parameter estimates $\hat{D}$ and $\hat{E}$, we compute

$$\mathbb{P}(C = k) \quad \text{for} \quad k = 0, \ldots, T-1,$$
$$\mathbb{P}(C \geq T) = 1 - \sum_{k=0}^{T-1} \mathbb{P}(C = k).$$

We then exploit that the uncensored mean (and similarly the variance) can be partitioned into a sum of the (scaled) censored mean and the mean (or variance) terms "missing" from the censored mean, i.e. we compute updated estimates $\hat{D}'$ of the uncensored mean and $\hat{v}'_u$ of the uncensored variance,

$$\hat{D}' := \mathbb{P}(C \geq T) \cdot \hat{m} + \sum_{k<T} k \cdot \mathbb{P}(C = k),$$
$$\hat{v}'_u := \mathbb{P}(C \geq T) \cdot (\hat{v} + \hat{m}^2) - \hat{D}'^2 + \sum_{k<T} k^2 \cdot \mathbb{P}(C = k).$$

Given the updated estimates of the uncensored moments, the updated reaction efficiency estimate $\hat{E}'$ is computed as in the case $T = 0$ as

$$\hat{E}' := \frac{1-r}{1+r} \quad \text{where} \quad r = \frac{\hat{v}'_u - \hat{D}'}{\hat{D}'^2} \quad \text{limited to } [0,1].$$

S1.3 Multiple initial copies

If each distinct molecules the sample initially contains $R > 1$ identical copies (e.g. $R = 2$ if the initial molecules are double-stranded), each of these copies can be imagined to be amplified by a separate and independent PCR processes. But since the molecules are indistinguishable, these processes cannot be observed individually – we can observe only the (re-normalized) sum of the resulting family sizes. These observed normalized family size distribution is thus the average of R independent versions of F, and its variance is thus one R-th of the variance in Equation (6), i.e.

$$\mathbb{V}F = \frac{1-E}{1+E} \cdot \frac{1}{R}, \quad \mathbb{V}C = D + D^2 \cdot \frac{1-E}{1+E} \cdot \frac{1}{R}. \qquad \text{(S1)}$$

The density of distribution of F for $R > 1$ is the R-fold self-convolution of the density of F with itself (re-scaled to again have expected value one), and can thus be computed from the pre-computed matrix for the single-molecule case without performing additional simulations.

Parameter estimation proceeds just as for $R = 1$, except that when computing estimate v' of $\mathbb{V}F$, we must now account for the reduction of the observed variance of F by a factor of $\frac{1}{R}$, i.e. we set $v' = R \cdot \frac{\hat{v}-\hat{m}}{\hat{m}^2}$.

S1.4 Data Analysis

The reads from each of the downloaded sequenced libraries, were mapped (ignoring the barcode part) with *NGM* v0.5.2 (Sedlazeck *et al.*, 2013) to the

© The Author(s) 2018.Published by Oxford University Press.

This is an Open Access article distributed under the terms of the Create Commons Attribution License (http://creativecommons.org/licenses/by/4.0), which permits unrestricted reuse, distribution, and reproduction in any medium, provided the original work is properly cited.

reference transcriptome of *D. melanogaster* (R6.08) respectively *E. coli* (strain K-12 MG1655). To avoid ambiguities during mapping for genes with multiple isoforms, we filtered the *D. melanogaster* transcriptome to contain only a single transcript per gene before mapping. For each gene, we picked either the single transcript with a FlyBase score of at least "moderately supported", or the longest transcript (if multiple ones had score "moderately supported" or higher). After mapping the reads, we used the combination of mapping coordinates (both start and end for the paired-end *E. coli* data, only start for the single-end *D. melanogaster* data) and barcode (on both ends in the case of *E. coli*) as UMI. To account for sequencing errors, we merged similar UMIs (barcodes differing at most in one position, mapping coordinates by at most 30 bases for paired-end, 5 for sing-end libraries) using the graph-based algorithm of Smith *et al.* (2017). For the *E. coli* data we additionally combined reciprocal UMIs stemming from the two strands of a single template molecule, but stored the read counts for plus- and minus-strand separately (see Shiroguchi *et al.* (2012)).

This yielded, for each of the libraries, a table comprising the gene id, start- end end position, barcode and read-count(s) of each detected UMI. Based on this table, the error-correction thresholds ($T = 5$ for *E. coli*, $T = 5$ for *D. melanogaster* R1, $T = 2$ for *D. melanogaster* R2), and the initial number of molecules (actually, strands) for each UMI ($R = 1$ for *E. coli* due to the Y-shaped adapters, $R = 2$ for *D. melanogaster* due to secondary strand synthesis before amplification) our algorithm computed library-wide and raw as well as shrunken gene-specific estimates of the reaction efficiency, of the average number of reads per UMI, and of the loss. For the *E. coli* data, the error-correction threshold was applied to the plus- and minus-strand read counts separately, filtering out UMIs if either count lay below the chosen threshold. This increased the loss of true UMIs, and we modified the definition of the loss accordingly to $\ell = 1 - (1 - \mathbb{P}(C < T))^2$ (compare to Equation (12)). (Note that in the histograms in Fig. 2A, for a lack of other options, we show plus- and minus-strand counts separately, but omit UMIs where one of the strands is not detected at all). In addition to the gene-specific parameter and loss estimates, our algorithm output the observed number of UMIs n_g^{obs} and the estimated total number of UMIs (i.e. transcript molecules) n_g^{tot}.

S1.5 Simulation

We determined the residual error of the corrected transcript counts using a simulation approach. We started from the (loss-corrected) estimated transcript counts n_g^{tot} and (shrunken) gene-specific estimates for reaction efficiency $\hat{E}_g$ and sequencing depth $\hat{D}_g$ of gene $g \in \{1, \ldots, K\}$ that we computed for replicate 1 of the *D. melanogaster* dataset. First we rounded n^{tot} to the next number in the series $10, 30, 100, 300, \ldots$ and used the resulting number as the *true* number n_g^{true} of transcripts of gene g. For each gene g, we then used the amplification+sequencing model (with parameters E_g, D_g and $R = 2$ meaning double-stranded molecules) to simulate the sequencing of n_g^{true} UMIs, which yielded for each gene n_g^{true} read counts, one for each UMI. To this list comprising gene id and (for each gene) n_g^{true} read counts, we applied our algorithm, using $T = 5$ and $R = 2$ as before (but passing along no other information from the first run of the algorithm). The algorithm thus dropped all UMIs with fewer than $T = 5$ reads, treated the remaining UMIs for each gene g as the *observed* number of UMIs n_g^{obs}, re-estimated the (shrunken) gene-specific losses, and used them to correct n_g^{obs} for these losses to arrive at an estimated total transcript count n_g^{tot}. Finally, we computed for each gene the *relative quantification error* as

$$\frac{\left|n_g^{\text{tot}} - n_g^{\text{true}}\right|}{n_g^{\text{true}}}. \tag{S2}$$

References

Akima, H. (1996). Algorithm 760; rectangular-grid-data surface fitting that has the accuracy of a bicubic polynomial. *ACM Transactions on Mathematical Software*, **22**(3), 357–361.

Sedlazeck, F. J., Rescheneder, P., and von Haeseler, A. (2013). NextGenMap: fast and accurate read mapping in highly polymorphic genomes. *Bioinformatics*, **29**(21), 2790–2791.

Shiroguchi, K., Jia, T. Z., Sims, P. A., and Xie, X. S. (2012). Digital RNA sequencing minimizes sequence-dependent bias and amplification noise with optimized single-molecule barcodes. *Proceedings of the National Academy of Sciences of the United States of America*, **109**(4), 1347–1352.

Smith, T., Heger, A., and Sudbery, I. (2017). UMI-tools: modeling sequencing errors in Unique Molecular Identifiers to improve quantification accuracy. *Genome research*, **27**(3), 491–499.

S2 Supplementary Figures

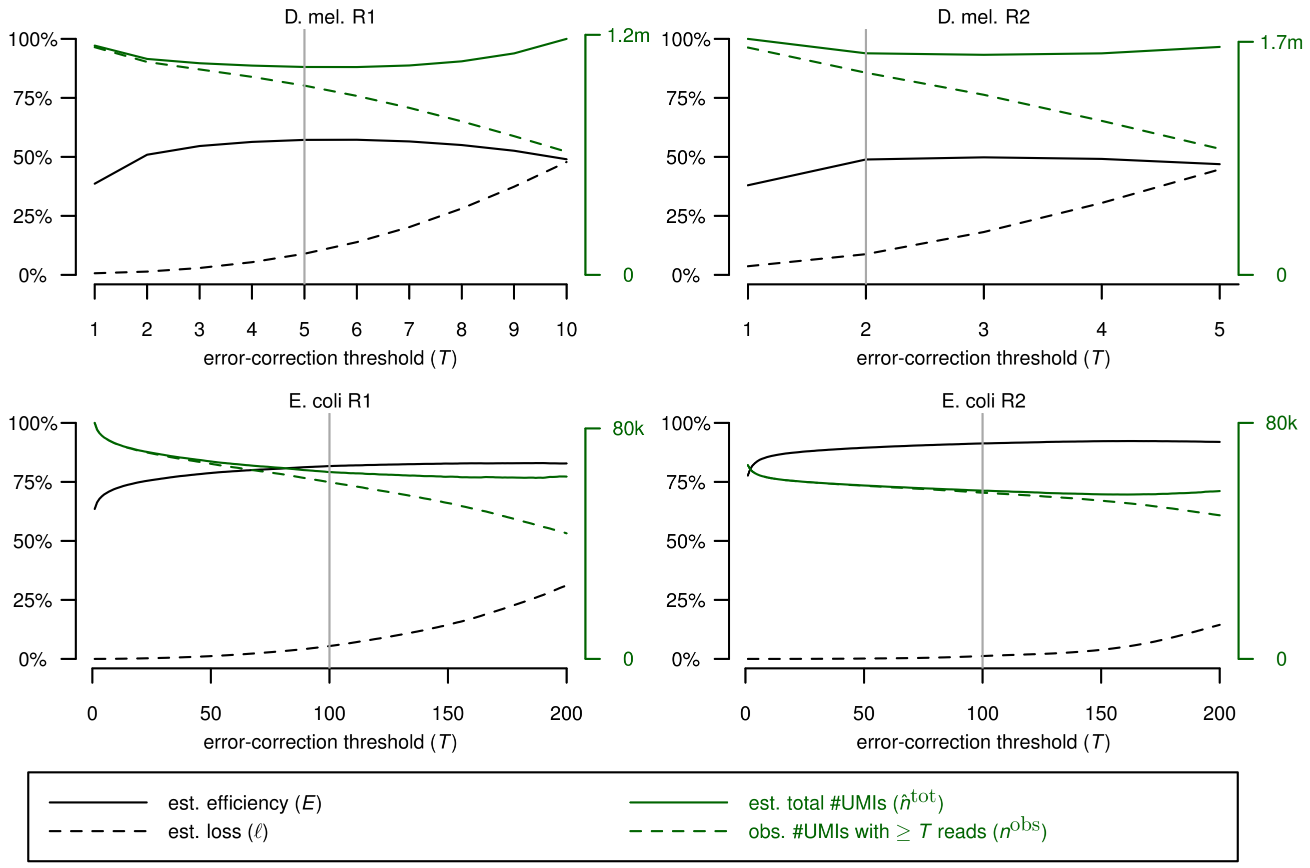

Fig. S1. Sensitivity of estimates to choice of threshold T. Shows the estimated efficiency (E, left y-axis), loss (ℓ, left y-axis), number of putative true UMIs (n^{obs}, right y-axis) and estimated total number of molecules ($\hat{n}^{\text{tot}}$, right y-axis) for different choices for the error-correction threshold T.

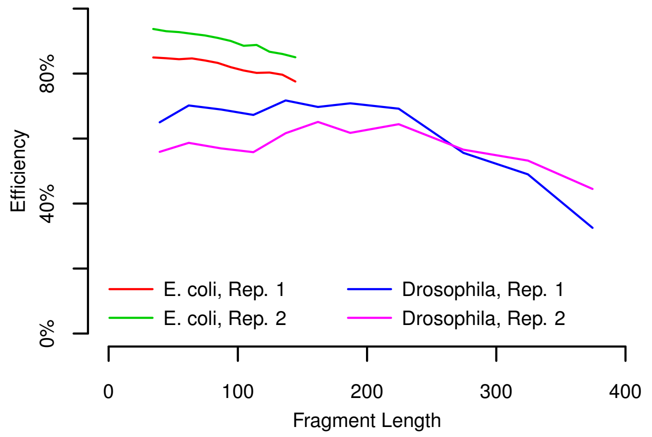

Fig. S2. Length dependence of PCR efficiency. For each experiment, the detected UMIs were binned according to fragment length, and the PCR efficiency estimated independently for each bin.

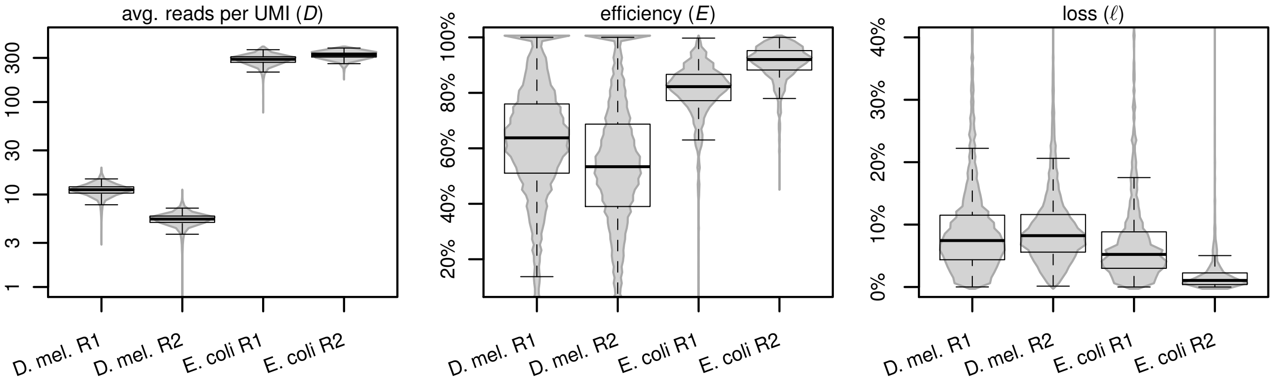

Fig. S3. Variability of the raw (unshrunken) model parameters and resulting loss between genes. Includes parameter for 7481 detected genes in D. mel. R1, 8001 genes in R2, 2380 genes in E. coli R1 and 2308 genes in R2.

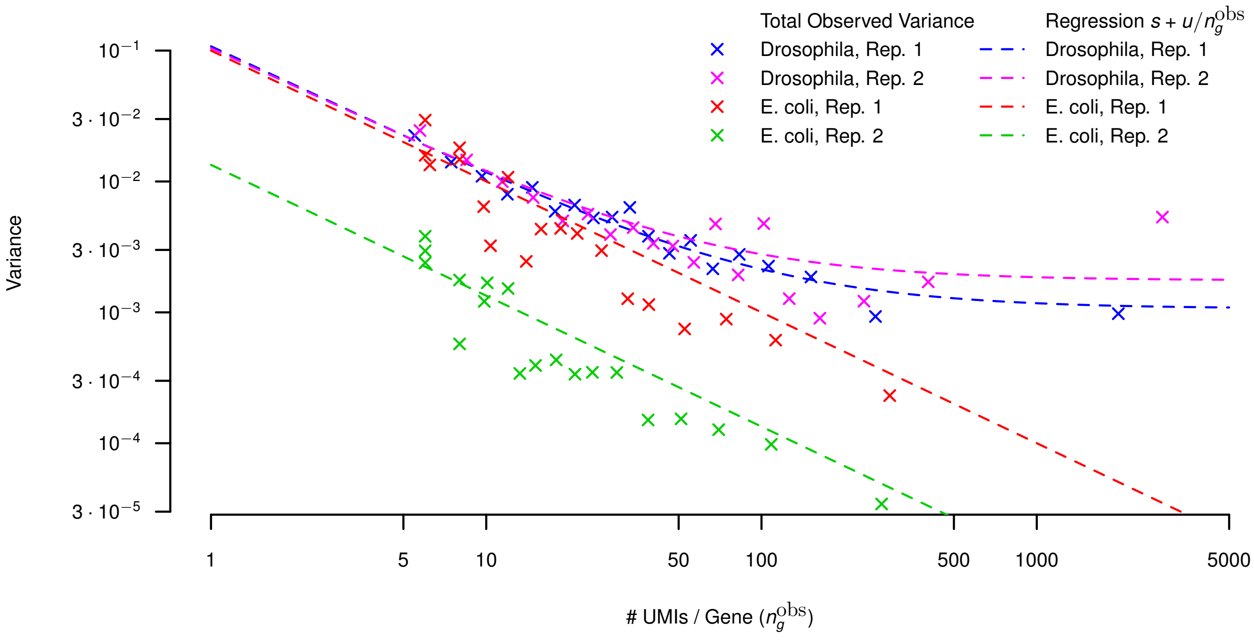

Fig. S4. Total variance of the raw gene-specific loss estimates. Total observed variance was computed for bins containing 20 genes with a similar number n_g^{obs} of observed true UMIs. The regression curve $s + u/n^{\mathrm{obs}}$ used to infer the optimal gene-specific shrinkage factors λ_g comprises two components, the variance s of the loss between genes, and the n_g^{obs}-dependent error of the (raw) gene-specific loss estimates u/n_g^{obs}. See also Gene-specific estimates & corrections.
